# High-Flow Nasal Cannula as an Alternate Weaning Strategy: A Randomized Controlled Trial

**DOI:** 10.7759/cureus.36511

**Published:** 2023-03-22

**Authors:** Gauri Arora, Zia Arshad, Ravi Prakash, Mudita Sharma, Gyan Prakash Singh, Monica Kohli

**Affiliations:** 1 Anesthesiology and Critical Care, King George’s Medical University, Lucknow, IND

**Keywords:** high-flow nasal cannula, spontaneous breathing trial, weaning failure, hap, weaning, sbt, hfnc

## Abstract

Background

Extubation failure is associated with increased morbidity and poor outcomes. This study aimed to ascertain the effectiveness of a high-flow nasal cannula (HFNC) as a weaning method compared to conventional weaning.

Methodology

A total of 60 mechanically ventilated patients, aged 18-65 years, who were ventilated for 48 hours and whose underlying pathology had either resolved or was improving, were enrolled in this study. They were randomized in a 1:1 ratio to participate in the HFNC weaning method or receive conventional weaning. Patients in Group A were extubated and oxygen was provided via HFNC. Group B patients were given a spontaneous breathing trial (SBT) per the standard protocol and extubated after a successful SBT.

Results

Weaning failure was found in five patients and was higher in the conventional group (three patients in the conventional group and two patients in the HFNC group). The duration of stay of patients in intensive care units was significantly higher in the conventional group than in the HFNC group.

Conclusions

HFNC is a better alternative to conventional weaning through SBT.

## Introduction

Extubation failure is associated with increased morbidity and poor outcomes. Re-intubation incidents have been reported to range between 10% and 20% after planned extubation and vary according to patient characteristics, weaning modalities, and follow-up periods [1]. There is a five-fold increase in the risk of mortality in patients with weaning failure. In addition, these patients have a prolonged intensive care unit (ICU) stay which increases morbidity and financial burden [2]. The beneficial effects of high-flow nasal cannula (HFNC) have been widely reported. Some of these include heated and humidified oxygen delivered during HFNC improves patient comfort; flushing of anatomical dead space lowers the CO2 level [3,4]; and the expiratory resistance produced by the continuous high flow causes a low level of positive oropharyngeal airway pressure [5]. HFNC systems are being increasingly utilized in critically ill infants, children, and adults. When compared to a regular nasal cannula and facemask oxygen, HFNC appears to provide an increased level of respiratory support. HFNC initially began as an alternative respiratory support to nasal continuous positive airway pressure (CPAP) for premature infants but is being increasingly utilized in patients with respiratory distress [4]. There is little published information on the use of HFNC as a weaning method in the Indian population. Well-designed trials are needed to create evidence-based data on the use of HFNC as a method of weaning in mechanically ventilated patients. Given this gap in knowledge, this study aimed to determine if planned extubation immediately followed by high-flow oxygen therapy would lower the need for re-intubation and help improve prognosis compared to conventional weaning in mechanically ventilated patients.

## Materials and methods

Ethical clearance for the study was given by King George’s Medical University Institutional Ethics Committee (human) (reference code: VI-PGTSC-IIA/P42). This was a randomized prospective controlled trial conducted in a tertiary care ICU. Because no Indian data are available on the use of HFNC for weaning, this is the first study of its kind in an Indian hospital setup. Therefore, a pilot sample of 60 patients (30 in each arm) was selected. The sample size was chosen based on the convenience and availability of patients and resources at the center where the study was conducted. This is an open-label study with no blinding.

After obtaining informed and written consent from patients’ attendants, 60 mechanically ventilated patients, aged 18-65 years, who were ventilated for >48 hours and whose underlying pathology had either resolved or was improving, were enrolled in this study. Patients needed to meet the weaning criteria. Patients with a history of cardiac arrest, acute coronary syndrome or life-threatening arrhythmias, failure of more than two organs, recent trauma or burns of the neck and face, unplanned or self-extubation, Acute Physiology and Chronic Health Evaluation (APACHE) score >25, once-failed weaning trial, tracheostomy, or neuromuscular disorder, as well as non-cooperating patients and pregnant women, were excluded.

Weaning criteria

Clinical Criteria

Clinical criteria included the initial reason for providing ventilatory support to be resolved or significantly improved, adequate hemoglobin (>8 mg/dL), stable hemodynamics (systolic blood pressure between 90 and 160 mmHg, diastolic blood pressure between 60 and 90 mmHg with minimal or no vasopressors, and heart rate ≤140 beats per minute), no evidence of myocardial ischemia or cardiac arrhythmias, no significant fever or infection, and arousable patient, and no significant electrolyte abnormality.

Ventilatory Parameters

Ventilatory parameters included a stable spontaneous ventilator drive, tidal volume of 4-6 mL/kg, and a rapid shallow breathing index <105.

Arterial Blood Gas Parameters

Arterial blood gas parameters were PaO_2_ >60 mmHg on FiO_2_ ≤50%, PaCO_2_ <50mmHg on FiO_2_ ≤50%, PaO_2_/FiO_2_ ratio of >200 mmHg, pH of 7.35-7.45, and HCO_3_ of 22-26 mEq/L [6].

Patients fulfilling the inclusion and exclusion criteria were randomized in a 1:1 ratio to participate in the HFNC weaning method or receive conventional weaning. Randomization was performed using a computer-generated list of random numbers. Treatments assigned were concealed in consecutively numbered, sealed, opaque envelopes, which were opened upon patient enrollment. Patients in group A were extubated, and oxygen was provided via HFNC. Patients in group B were given a spontaneous breathing trial (SBT) per the standard protocol and extubated after a successful SBT.

SBT was considered successful if the respiratory rate was <35 breaths per minute, heart rate was <140 beats per minute, heart rate variability was >20%, arterial oxygen saturation was >90% or pO_2_ >60 mmHg on 40% FiO_2_, systolic blood pressure was within 100-180 mmHg, and there were no signs of breathing difficulty or distress [6].

HFNC failure was predicted using the ROX criteria (ratio of pulse oximetry/FiO_2_ to respiratory rate) [7].

**Figure 1:**
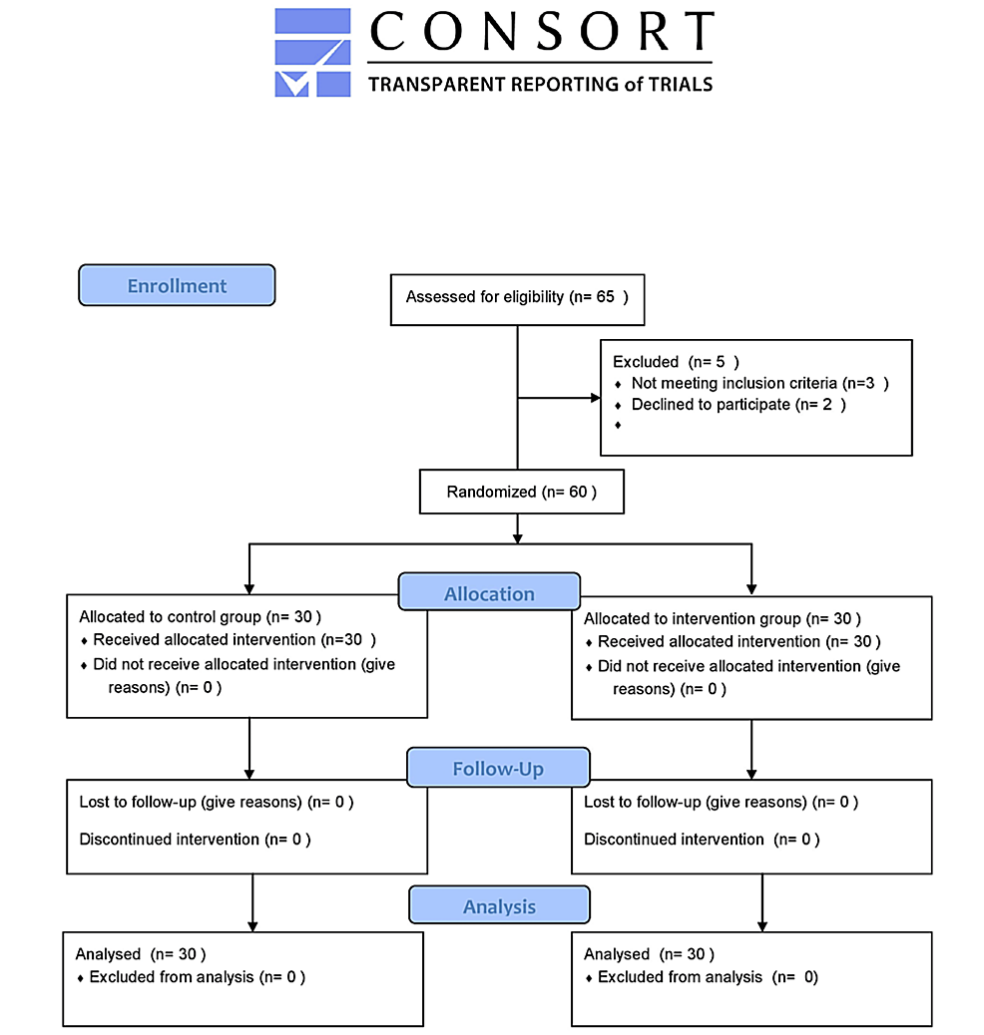
Consort flow diagram.

After successful extubation, patients were evaluated daily by the in charge of the ICU (not part of the study) and shifted out of the ICU when vital functions were found to be stable without the need for support and did not require further monitoring or treatment [8]. Figure 1 shows the Consort flow diagram.

## Results

After satisfying the weaning criteria, 60 patients on mechanical ventilation were included in the study and equally divided into two groups (n = 30). The percentage of female patients was 71.7%, and that of males was 28.3%. Females were the majority in both groups, and the association of distribution of patients was found to be statistically non-significant (p > 0.05). The mean age of patients of group A was 35.63 years, and that of group B was 32.50. The difference among them was found to be statistically non-significant (p > 0.05). The mean APACHE score was lower in group A (13.87 ± 2.57) than in group B (14.43 ± 2.87), and the difference among them was found to be statistically non-significant (p > 0.05) (Table 1).

**Table 1:** Distribution of patients based on gender, age, and APACHE score. APACHE: Acute Physiology and Chronic Health Evaluation

		Group		Total	P-value
		Group A (n = 30)	Group B (n = 30)		
Gender	Male	9 (30.0%)	8 (26.7%)	17 (28.3%)	0.500
	Female	21 (70.0%)	22 (73.3%)	43 (71.7%)	
Age (in years)		35.63 ± 15.32	32.50 ± 12.80	34.07 ± 14.08	0.394
APACHE score		13.87 ± 2.57	14.43 ± 2.87	14.15 ± 2.71	0.423

Ventilator and total ICU days were significantly lower for patients in group A than in group B (p < 0.05), but only ICU stay was similar and statistically non-significant (p > 0.05).

Weaning failure was found in a total of five patients and was higher in group B (3; 10.0%) than in group A (2; 6.7%). The association among them was found to be statistically non-significant (p > 0.05).

Incidence of hospital-acquired pneumonia was found in three patients, and it was higher in group B (3; 10%) than in group A (0%). The association among them was found to be statistically non-significant (p > 0.05) (Table 2).

**Table 2:** Comparison of ICU stay, weaning failure, hours on the ventilator, and HAP in both groups. ICU: intensive care unit; HAP: hospital-acquired pneumonia

	Group		Total (n = 60), mean ± SD	P-value
	Group A (n = 30), mean ± SD	Group B (n = 30), mean ± SD		
ICU days (after extubation)	2.95 ± 1.27	3.63 ± 2.26	3.29 ± 1.86	0.155
Total ICU stay (in hours)	97.87 ± 34.99	125.33 ± 56.27	111.60 ± 48.48	0.027
Weaning failure	2 (6.7%)	3 (10.0%)	5 (8.3%)	0.640
Hours on ventilator	74.17 ± 32.78	95.23 ± 38.28	83.47 ± 37.56	0.026
HAP	0 (0%)	3 (10%)	3 (5.0%)	0.7

## Discussion

Oxygenation impairment in the period following planned extubation is common and usually rectified by providing oxygen through nasal prongs or Venturi masks, with a fraction of inspired oxygen (FiO_2_) and flow depending on the degree of hypoxemia [9]. Acute respiratory failure is the most common cause of the need for re-intubation in critically ill patients. It was estimated that 40-65% of patients in ICUs needed mechanical ventilation [10], which provided adequate oxygenation and reduced the work of breathing for patients with respiratory failure due to different etiologies [11]. Weaning covers the entire process of liberating the patient from mechanical support and the endotracheal tube. Complications of mechanical ventilation include respiratory muscle weakness, upper airway injury, ventilator‐associated pneumonia (VAP), sinusitis, and associated death [12].

Technological improvements have made it possible for high-flow oxygen therapy to be delivered via nasal cannula, which allows constant FiO_2_ during peak inspiratory flow and has the added advantage of a low level of continuous positive airway pressure [13] with high end-expiratory lung volume [14] and decreased work of breathing by intrinsic positive end-expiration pressure compensation and dead space washout [4]. HFNC provides warmed and humidified inspired gases, thus improving comfort and possibly reducing airway inflammation [15], helping with better drainage of secretions in the respiratory tract [16].

Clinical research studies conducted in severely ill patients have concluded that high-flow oxygen given during acute respiratory failure boosts oxygenation, survival [17], tolerance, and comfort and eases respiratory secretion drainage [18]. The proportion of patients who were re-intubated for non-respiratory-related causes depends on the case mix, which differs widely among ICUs and has not usually been reported in research studies. In a study, approximately 30% of re-intubations were related to non-respiratory causes as the case mix included a higher number of postsurgical and critical neurological patients. After excluding these causes, the re-intubation rate in this study was comparable to that expected in a low-risk population. APACHE II is a system to classify disease severity, which is frequently applied to predict the risk of death for critically ill patients. It has been reported that patients with >15 APACHE II scores have a greater risk of nosocomial infection and death [19]. In our study, although the difference in the rate of need for re-intubation in patients weaned by HFNC compared to conventional weaning was not statistically significant, the patients who were weaned by HFNC showed a decrease in the duration of total ICU stay and mechanical ventilation requirement. Weaning failure was noted in five patients. It was higher in the conventional group than in the HFNC group, and the association among them was found to be statistically non-significant. Patients who have difficulty in weaning off from mechanical ventilation tend to have a prolonged ICU stay, which is associated with muscle atrophy and decreased muscle strength.

Once these patients have recovered from a critical illness, they usually experience significantly reduced functional ability and a lower quality of life, requiring a long period of rehabilitation. HFNC supports the respiratory system after extubation effectively, and this is largely due to the high flow of the administered gas, which can meet the ventilator demand of the patient after extubation. Re-intubation due to post-extubation respiratory failure is associated with increased duration of ICU stay, hospital stay, and mortality [20].

In a similar study, Maggiore et al. randomized a sample of critically ill patients to either be administered high-flow oxygen therapy or conventional oxygen therapy for 48 hours. The results showed significantly higher oxygenation in the high-flow oxygen group, along with a lower re-intubation rate of 3.8%. They also concluded that high-flow oxygen improved the drainage of respiratory secretions, helping in lowering the rate of re-intubation [16].

Mungan et al. conducted a prospective, observational, cohort study to determine the effects of the use of HFNC, conventional oxygen therapy, and non-invasive ventilation (NIV) on weaning failure in high-risk patients and recorded the lowest re-intubation rate along with a reduced ICU stay and hospital stay in the HFNC group. The study also reported that the total mechanical ventilation assistance duration and length of stay in the ICU and hospital differed between groups receiving NIV, HFNC, and conventional oxygen therapy, thus favoring the high-flow oxygen therapy group [21].

Hernandez et al. conducted a study among patients who successfully passed SBT and were at a lower risk for re-intubation and recorded fewer re-intubation numbers within 72 hours with the use of HFNC (4.9% needed re-intubation) than with conventional oxygen therapy (12.2%) [9].

Fernandez et al. reported that HFNC decreases post-extubation respiratory failure in high-risk non-hypercapnic patients compared with conventional oxygen therapy [22]. Zhu et al. concluded that compared with conventional oxygen therapy, HFNC may notably lower post-extubation respiratory failure and respiratory rates, improve PaO_2_, and can be safely administered in patients after planned extubation [23].

In a study by Frat et al., in patients with PaO_2_/FiO_2_ <300, the use of HFNC at 50 L/minute was more successful in ventilating patients compared to conventional oxygen therapy or NIV, with fewer need for intubation in the HFNC group (38%) compared to NIV (50%) and conventional oxygen therapy (47%) [17].

A comparison of ventilation and ICU stay duration between both groups revealed a higher duration of mechanical ventilation dependence as well as ICU stay in the conventional group compared to the HFNC group.

Cho et al. reported that the duration of ICU stay post-weaning did not differ between the groups, which is in accordance with this study where ICU stay post-weaning did not differ significantly between groups, although the difference in total ICU stay was significant. [24].

VAP is the most frequent infection in ICUs. *Acinetobacter baumannii* and *Pseudomonas aeruginosa*, which particularly resist carbapenem antibiotics, are the most common pathogens causing VAP or nosocomial respiratory infections and may pose important therapeutic challenges [25]. Patients in ICUs often receive broad-spectrum antibiotic treatment. We observed hospital-acquired respiratory infections in three cases in the conventional group during the weaning period compared to none in the HFNC group.

Among patients who were extubated and were at low risk for re-intubation, the use of HFNC, compared with conventional weaning, proved to be an acceptable alternate weaning method. HFNC supports the respiratory system effectively after extubation, which is largely due to the high flow of the administered gas, which can meet the ventilator demand of the patient after extubation. If an effective combination of these techniques is applied, it might significantly help in the treatment of patients who are difficult to wean and are at a high risk of re-intubation. In times of financial crisis, it is crucial to find and establish cost-effective procedures. It is well documented that reducing the length of ICU stay and minimizing its side effects could have significant economic benefits on health systems worldwide. Such methods also prove highly effective in reducing the burden on ICUs for the availability of resources and ventilators for the management of critically ill patients.

Our study has some limitations. This was a single-center study conducted among very few patients. A smaller sample size limited our statistical power to detect differences in some parameters, leading to wide confidence intervals. Due to the limitation of resources, other variables such as forced vital capacity, negative inspiratory force, and Vd/Vt ratio (dead space volume and tidal volume ratio) could not be included. Implementing a more complex weaning protocol would have been a better predictor in analyzing the correct time to start weaning. Comparison of more dynamic parameters at different intervals can yield additional information.

## Conclusions

Our study concluded that there is a significant decrease in the duration of ICU stay and mechanical ventilation in patients who were administered HFNC as a weaning strategy compared to those who were weaned conventionally using either T-piece or CPAP mode. The patients weaned with HFNC were more comfortable, with a less invasive method of ventilation. It also decreased the workload on ICU staff.

The study established that in selected populations, HFNC can be used as efficiently as other weaning strategies. Hence, we conclude that HFNC is an equally effective method of weaning as conventional weaning. However, the familiarity of the intensivist and their experience with HFNC play a crucial role.

## References

[1] Gowardman JR, Huntington D, Whiting J (2006). The effect of extubation failure on outcome in a multidisciplinary Australian intensive care unit. Crit Care Resusc.

[2] Menon N, Joffe AM, Deem S (2012). Occurrence and complications of tracheal reintubation in critically ill adults. Respir Care.

[3] Dysart K, Miller TL, Wolfson MR, Shaffer TH (2009). Research in high flow therapy: mechanisms of action. Respir Med.

[4] Lee JH, Rehder KJ, Williford L, Cheifetz IM, Turner DA (2013). Use of high flow nasal cannula in critically ill infants, children, and adults: a critical review of the literature. Intensive Care Med.

[5] Parke RL, Eccleston ML, McGuinness SP (2011). The effects of flow on airway pressure during nasal high-flow oxygen therapy. Respir Care.

[6] Zein H, Baratloo A, Negida A, Safari S (2016). Ventilator weaning and spontaneous breathing trials; an educational review. Emerg (Tehran).

[7] Junhai Z, Jing Y, Beibei C, Li L (2022). The value of ROX index in predicting the outcome of high flow nasal cannula: a systematic review and meta-analysis. Respir Res.

[8] Bakker J, Damen J, van Zanten AR, Hubben JH (2003). [Admission and discharge criteria for intensive care departments]. Ned Tijdschr Geneeskd.

[9] Hernández G, Vaquero C, González P (2016). Effect of postextubation high-flow nasal cannula vs conventional oxygen therapy on reintubation in low-risk patients: a randomized clinical trial. JAMA.

[10] Linko R, Okkonen M, Pettilä V (2009). Acute respiratory failure in intensive care units. FINNALI: a prospective cohort study. Intensive Care Med.

[11] Coursin DB (1999). Physiological basis of ventilatory support. Anesthesiology.

[12] Burns KE, Meade MO, Premji A, Adhikari NK (2013). Noninvasive positive-pressure ventilation as a weaning strategy for intubated adults with respiratory failure. Cochrane Database Syst Rev.

[13] Parke RL, McGuinness SP (2013). Pressures delivered by nasal high flow oxygen during all phases of the respiratory cycle. Respir Care.

[14] Riera J, Pérez P, Cortés J, Roca O, Masclans JR, Rello J (2013). Effect of high-flow nasal cannula and body position on end-expiratory lung volume: a cohort study using electrical impedance tomography. Respir Care.

[15] Roca O, Riera J, Torres F, Masclans JR (2010). High-flow oxygen therapy in acute respiratory failure. Respir Care.

[16] Maggiore SM, Idone FA, Vaschetto R (2014). Nasal high-flow versus Venturi mask oxygen therapy after extubation. Effects on oxygenation, comfort, and clinical outcome. Am J Respir Crit Care Med.

[17] Frat JP, Thille AW, Mercat A (2015). High-flow oxygen through nasal cannula in acute hypoxemic respiratory failure. N Engl J Med.

[18] Kilgour E, Rankin N, Ryan S, Pack R (2004). Mucociliary function deteriorates in the clinical range of inspired air temperature and humidity. Intensive Care Med.

[19] Ma W, Wang L, Zhang JL (2010). Relevance between APACHE II score and nosocomial infection in ICU patients: a comparative study. Chin J Nosocomiol.

[20] Mauri T, Turrini C, Eronia N, Grasselli G, Volta CA, Bellani G, Pesenti A (2017). Physiologic effects of high-flow nasal cannula in acute hypoxemic respiratory failure. Am J Respir Crit Care Med.

[21] Mungan I, Turan S (2021). The comparison of non-invasive ventilation, high-flow oxygen therapy and conventional oxygen therapy for weaning failure in high-risk patients. GKDA Derg.

[22] Fernandez R, Subira C, Frutos-Vivar F (2017). High-flow nasal cannula to prevent postextubation respiratory failure in high-risk non-hypercapnic patients: a randomized multicenter trial. Ann Intensive Care.

[23] Zhu Y, Yin H, Zhang R, Ye X, Wei J (2019). High-flow nasal cannula oxygen therapy versus conventional oxygen therapy in patients after planned extubation: a systematic review and meta-analysis. Crit Care.

[24] Cho JY, Kim HS, Kang H, Kim SH, Choe KH, Lee KM, Shin YM (2020). Comparison of postextubation outcomes associated with high-flow nasal cannula vs. conventional oxygen therapy in patients at high risk of reintubation: a randomized clinical trial. J Korean Med Sci.

[25] Esteban A, Anzueto A, Alía I (2000). How is mechanical ventilation employed in the intensive care unit? An international utilization review. Am J Respir Crit Care Med.

